# Effects of Retinal Transcription Regulation After GB20 Needling Treatment in Retina With Optic Neuritis

**DOI:** 10.3389/fnint.2020.568449

**Published:** 2020-09-29

**Authors:** Jie Chen, Li Zhang, Xiulun Gan, Rong Zhang, Yinjia He, Qiuyi Lv, Haonan Fu, Xiaodong Liu, Linqing Miao

**Affiliations:** ^1^Beijing Advanced Innovation Center for Intelligent Robots and Systems, Beijing Institute of Technology, Beijing, China; ^2^School of Acupuncture-Moxibustion and Tuina, Beijing University of Chinese Medicine, Beijing, China; ^3^School of Traditional Chinese Medicine, Beijing University of Chinese Medicine, Beijing, China; ^4^School of Mechatronical Engineering, Beijing Institute of Technology, Beijing, China

**Keywords:** needling, GB20 (Fengchi), GV16 (Fengfu), RNA sequencing, optic neuritis

## Abstract

Optic neuritis (ON) is one of the most frequent symptoms of multiple sclerosis (MS) that results in progressive loss of axons and neurons. In clinical trials of Traditional Chinese Medicine, needling at the GB20 acupoint has been widely used for the treatment of ocular diseases, including ON. However, the molecular mechanisms of needling at this site are still unclear. In this study, we generated an experimental autoimmune encephalomyelitis (EAE) mouse model and investigated the effects of needling treatment at the GB20 acupoint on retina with EAE-associated ON. RNA sequencing of the retinal transcriptome revealed that, of the 234 differentially expressed genes induced by ON, 100 genes were upregulated, and 134 genes were downregulated by ON, while needling at the GB20 acupoint specifically reversed the expression of 21 genes compared with control treatment at GV16 acupoint. Among the reversed genes, Nr4a3, Sncg, Uchl1, and Tppp3 were involved in axon development and regeneration and were downregulated by ON, indicating the beneficial effect of needling at GB20. Further gene ontology (GO) enrichment analysis revealed that needling at GB20 affected the molecular process of Circadian rhythm in mouse retina with ON. Our study first reported that needling treatment after ON at the GB20 acupoint regulated gene expression of the retina and reversed the expression of downregulated axon development-related genes. This study also demonstrated that GV16 was a perfect control treatment site for GB20 in animal research. Our study provided a scientific basis for needling treatments at GB20 for ocular diseases.

## Introduction

Multiple sclerosis (MS) is one of the most common inflammatory demyelinating diseases of the central nervous system, characterized by inflammation, demyelination, axonal loss, and gliosis. In MS patients, inflammation predominately affects the white matter of the brain and spinal cord, and it leads to typical presenting syndromes, including monocular visual loss, limb weakness, sensory loss, double vision, and ataxia, depending on the location of the MS lesions (Lassmann, 2018, 2019; Reich et al., 2018). As a major target in MS, the optic nerve axon could be easily injured, resulting in optic neuritis (ON). ON is a common manifestation and the second most frequent symptom of MS (Kemenyova et al., 2014). It mainly occurs in the setting of MS, and patients with ON typically show a progressive unilateral visual loss of variable severity (de Seze, 2013). The underlying mechanisms of MS-associated ON remain unclear, and there is no satisfactory treatment that could fully prevent visual disability (Woung et al., 2011). In some patients with typical demyelinating ON, no treatment is required, and visual loss is expected to recover from relapses spontaneously (Jenkins and Toosy, 2017). However, most relapses leave behind such persistent damage as color vision, contrast sensitivity, and depth perception abnormalities after the recovery of visual acuity (Dobson and Giovannoni, 2019). Evidence has demonstrated that steroid treatment could accelerate visual recovery but could not improve final visual outcome (Brusaferri and Candelise, 2000). In addition, for patients who fail to recover, no suitable therapy is available.

Needling is a kind of mechanical stimulation and a prevalent clinical methodology based on Traditional Chinese Medicine, which presents great therapeutic effects on ocular diseases without many side-effects. It has been used for treatment of ocular diseases like glaucoma, age-related macular degeneration, retinitis pigmentosa, et cetera (Jiao, 2011; Xu et al., 2012; Law and Li, 2013; Xu and Peng, 2015). According to Traditional Chinese Medicine, treatment sites of GB20 and BL1 acupoint are widely used for ON therapy. GB20 and BL1 were also selected for needling treatment of other ocular diseases (Xu and Peng, 2015; Qin et al., 2015). Our previous study showed that GB20 was more suitable than BL1 in animal study of ocular diseases (Chen et al., 2019). Although the application of needling has shown good clinical efficacy in the treatment of MS-associated ON, the mechanisms of needling are totally unknown. In this study, we use the experimental autoimmune encephalomyelitis (EAE)-optic neuritis (EAE-ON) model to elucidate the scientific basis of needling treatment of ON. EAE is a suitable model, in which animals develop inflammatory-demyelinating diseases spontaneously and cover the specific spectrum of the pathological and immunological features of MS (Lassmann and Bradl, 2017). In mice, the EAE-ON model is induced by injection of myelin oligodendrocyte glycoprotein (MOG) peptide-MOG35–55, and clinical EAE scores are graded daily and blindly according to the standard scoring system (Dietrich et al., 2018; Locri et al., 2018; Torre-Fuentes et al., 2020). Our previous results showed that retinal ganglion cells (RGC), in the retina, were most affected by ON (Huang et al., 2017). We also found that needling at GB20 increased RGC survival in an optic nerve crush model (Chen et al., 2019). However, the effect of needing at GB20 on retina with ON is still unclear. RNA sequencing (RNA-seq) is a new technique effective in identifying numerous genes regulated by specific treatment. Therefore, we take advantage of RNA-seq technique and the EAE mouse model to identify and analyze the differentially expressed retinal genes induced by inflammatory demyelination and the genes reversed after needling treatment at GB20 and reveal the regulation effects of needling treatment on the retina with ON.

## Materials and Methods

### Animals

We perform experiments on 8-week-old female C57BL/6 mice. All animal procedures were performed in accordance with the National Institute of Health guidelines. The protocol was approved by the Animal Care and Use Committee of Beijing Institute of Technology and Peking University.

#### MOG Immunization and EAE Model Preparation

Female 8-week-old C57BL/6 mice were immunized with 150 μg MOG35–55 peptide emulsified with complete Freund’s adjuvant (CFA) and 2.5 mg/ml mycobacterium tuberculosis, followed by immunization of 200 ng pertussis toxin at day 0 and day 2 (Quinn et al., 2011). The behavioral deficits of these mice were assessed daily with a 5-point scale (Gran et al., 2002), as follows: limp tail, 1; limp tail with waddling gait, 1.5; partial limb paralysis, 2; full paralysis of one limb, 2.5; full paralysis of one limb with partial paralysis of second limb, 3; full paralysis of two limbs, 3.5; moribund, 4; and death, 5. The clinical score was recorded every other day until 5 weeks post-immunization.

#### Needling Treatment

Three weeks after MOG immunization, mice were anesthetized by xylazine and ketamine based on their body weight (0.01 mg xylazine/g + 0.08 mg ketamine/g) before needling treatment at acupoint GB20 or GV16 at both sides, respectively. The depth of the needling is around 2 mm. The duration of needling treatment was 20 min. EAE mice were treated with needling treatments 3 weeks after MOG immunization and treated every 3 days for 2 weeks before sacrifice.

#### RNA Preparation

Mice were randomly divided into four groups (two or three mice/group). Experiments were repeated for three times. Briefly, in each replicate, mice were sacrificed 5 weeks after MOG immunization, and retinas were dissected out in HBSS buffer (Cellgro) immediately. Retinas were then homogenized with TRIzol Reagent (Thermo Fisher Scientific), and total RNA was extracted from the homogenized mixture according to the reagent instructions. RNA purity was checked using the NanoPhotometer^®^ spectrophotometer (IMPLEN). RNA concentration was measured using Qubit^®^ RNA Assay Kit in Qubit^®^ 2.0 Flurometer (Life Technologies). RNA integrity was assessed using the RNA Nano 6000 Assay Kit of the Bioanalyzer 2100 system (Agilent Technologies).

### Library Preparation and Sequencing

About 5 μg total RNA generated from each group was used for RNA-seq, which was done at Novogene, Inc. Briefly, RNA samples from three biological replicates went through mRNA purification with poly-T oligo-attached magnetic beads. Sequencing libraries were generated using NEBNext^®^ Ultra™ RNA Library Prep Kit for Illumina^®^ (NEB) following manufacturer’s recommendations and index codes were added to attribute sequences to each sample. The library fragments were purified with the AMPure XP system (Beckman Coulter) for cDNA fragments of preferentially 250–300 bp in length. Library quality was assessed on the Agilent Bioanalyzer 2100 system. The clustering of the index-coded samples was performed on a cBot Cluster Generation System using TruSeq PE Cluster Kit v3-cBot-HS (Illumia) according to the manufacturer’s instructions. After cluster generation, the library preparations were sequenced on an Illumina Hiseq platform and 125/150 bp paired-end reads were generated.

### Gene Expression Analysis

The RNA-seq reads were aligned to the reference genome using Hisat2 v2.0.5. FeatureCounts v1.5.0-p3 was used to count the reads numbers mapped to each gene. We used Fragments Per Kilobase of transcript sequence per Millions base pairs sequenced (FPKM) to represent relative gene expression abundance. FPKM of each gene was calculated based on the length of the gene and reads count mapped to this gene, which normalizes gene expression by considering the effect of sequencing depth and gene transcript length at the same time. Differential expression analysis was performed using the DESeq2 R package (1.16.1). The resulting *p*-values were adjusted using the Benjamini and Hochberg’s approach for controlling the false discovery rate (less than 0.05). Genes with an adjusted *p*-value (*p*adj) < 0.05 (detected by DESeq2) were considered differentially expressed.

### Statistical Analysis

An adjusted *p*-value <0.05 was considered as statistically significant. The raw data and GEO accession number for this study are as follows: GSE148759, link: https://www.ncbi.nlm.nih.gov/geo/query/acc.cgi?acc=GSE148759.

## Results

### Expression Analysis

**Figure 1:**
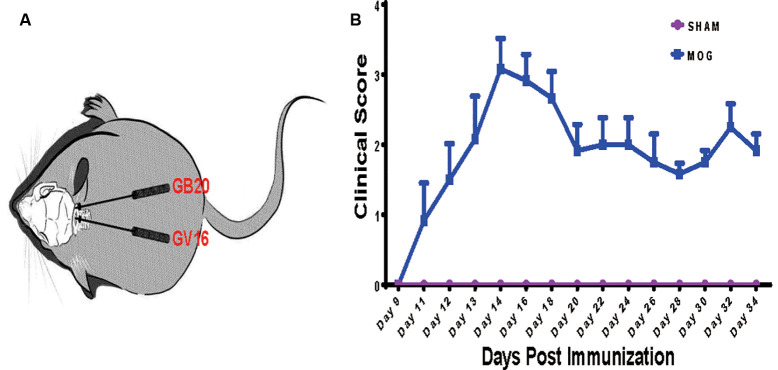
Needling treatment sites and experimental autoimmune encephalomyelitis (EAE) mice scoring. **(A)** Schematic illustration of needling at sites of GB20 and GV16 of the mouse. **(B)** Clinical scores of mice after MOG35–55 peptide immunization.

To investigate the effects of needling treatments on regulation of retinal gene expression in EAE/ON models, we extracted retinal RNA 5 weeks after immunization, from SHAM control group (SHAM), MOG immunization group (MOG) and MOG immunization mice with needling treatments at acupoint GB20 (GB20) or control acupoint GV16 (GV16). The schematic image illustrated the needling treatment sites of GB20 and GV16 (Figure 1A). Scores on a five-point scale showed the clinical development of mice after MOG immunization (Figure 1B). Needling treatment at GB20 or GV16 did not affect the EAE clinical score (data not shown).

**Figure 2:**
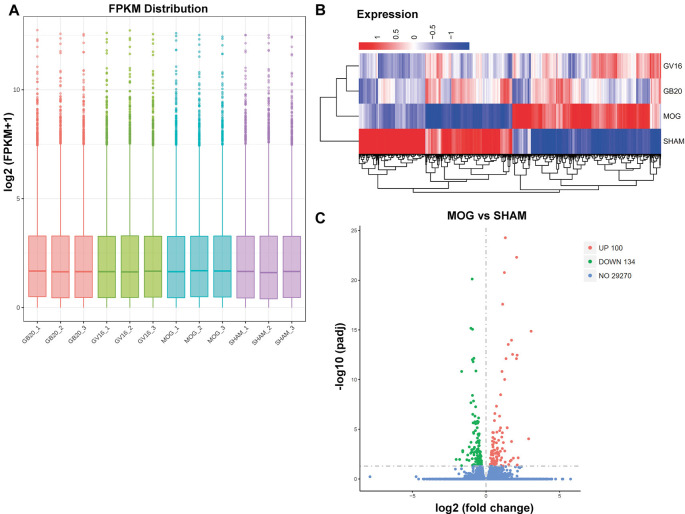
Quantitative analysis of retinal gene expression. **(A)** Boxplot shows the overall range and distribution of fragments per kilo base per million (FPKM) value of gene expression. **(B)** Cluster analysis of differentially expressed genes among groups. The colors of the heat map indicate the relative gene expression. Red color indicates the higher gene expression, while blue color indicates the lower gene expression. **(C)** The volcano map shows the numbers of differentially expressed genes (DEGs) of retina after myelin oligodendrocyte glycoprotein (MOG) immunization, compared with SHAM control. *p*adj: adjusted *p*-value.

RNA integrity of each sample was assessed by Bioanalyzer 2100 system. Sample total reads ranged from 44.4 to 54.7 million, and the mapping rate of sample total reads to mouse reference genome ranged from 97.0 to 97.3%. We used FPKM to represent relative gene expression abundance. The boxplot result showed that the overall distribution of the FPKM values were consistent among samples, suggesting that the RNA-seq data were reproducible (Figure 2A). Cluster analysis showed that needling treatment groups were clustered together and separated from MOG control, and all the MOG groups with or without needling treatments were separated from SHAM control group (Figure 2B).

### Differentially Expressed Retinal Genes by MOG Immunization

To identify candidate genes affected by optic neuritis, we performed differential expression analysis using the DESeq2 R package (1.16.1). Genes with |log2FoldChange| > 0 and an adjusted *p*-value <0.05 were assigned as differentially expressed. We then identified 234 differentially expressed genes (DEGs) in total, with 100 genes upregulated and 134 genes down-regulated (Figure 2C). The top most upregulated gene, Ecel1, had an alternative name “damage-induced neuronal endopeptidase,” whose molecular functions were metal ion binding and metalloendopeptidase activity (Kiryu-Seo et al., 2000). Ecel1 was increased by eight times after ON and the *p*-value was 1.41 × 10^−15^, which was incredibly significant. The second most upregulated gene, Oas2, was an interferon-induced dsRNA-activated antiviral enzyme that played a critical role in cellular innate antiviral response (Oakes et al., 2017). It also played a role in other cellular processes such as apoptosis, cell growth, differentiation and gene regulation (Kristiansen et al., 2011). Oas2 was increased by seven times after ON and the *p-value* was 9.17 × 10^−5^. The third most up-regulated gene was unknown. The fourth most upregulated gene, Cxcl10, was a pro-inflammatory cytokine that was involved in processes such as activation of peripheral immune cells, regulation of cell growth, apoptosis and modulation of angiostatic effects (Gao et al., 2017). Activation of the CXCL10/CXCR3 axis also played an important role in neurons in response to brain injury (Rappert et al., 2004). Cxcl10 was increased by four times after ON and the *p-value* was 0.0389. The fifth most upregulated gene, Tnnt2, whose molecular functions were actin binding and calcium ion binding, was increased by four times after ON and the *p-value* was 3.61 × 10^−13^. The top most down-regulated gene, Ppp1r1c, was a protein phosphatase inhibitor, increasing cell susceptibility to TNF-induced apoptosis. Ppp1r1c was down-regulated by 75% after ON and the *p*-value was 0.0107. The second most down-regulated gene, Oxtr, was a G-protein coupled receptor involved in many biological processes such as positive regulation of synapse assembly and synaptic transmission. Oxtr was down-regulated by 71% after ON and the *p*-value was 0.011. The third most down-regulated gene, Egr4, was a transcriptional regulator, activating the transcription of target genes required for mitogenesis and differentiation. Egr4 was down-regulated by 68% after ON and the *p*-value was 4.49 × 10^−2^. The fourth most down-regulated gene, Scn4b, was a voltage-gated sodium channel subunit, positively regulating sodium ion transport. Scn4b was down-regulated by 68% after ON and the *p*-value was 1.57 × 10^−11^. The fifth most down-regulated gene was an unknown gene. The top 20 upregulated and down-regulated DEGs together with the information of log2 FoldChange and *p*-value were listed in Table 1. Among the 20 down-regulated DEGs, there were four transcription factors, Egr4, Nr4a3, Isl2, and Pou4f1, which were highlighted in yellow (Table 1). The transcription factor Nr4a3 functioned in negative regulation of apoptotic process, and was reported to play a neuroprotection role in oxidative stress-induced neuron death (Rappert et al., 2004). Nr4a3 was down-regulated by 67% after ON and the *p*-value was 0.00183. The transcription factor Isl2, was reported to be involved in axonogenesis and retinal ganglion cell axon guidance (Pak et al., 2004). Isl2 was down-regulated by 55% after ON, and the *p*-value was 0.000594. The transcription factor Pou4f1, also named Brn3a, was another negative regulator of apoptotic process (Hudson et al., 2008; Dykes et al., 2010). Pou4f1 was down-regulated by 51% after ON and the *p-value* was 2.16 × 10^−8^.

**Table 1:** Top 20 upregulated and 20 down-regulated retinal genes induced by optic neuritis (ON).

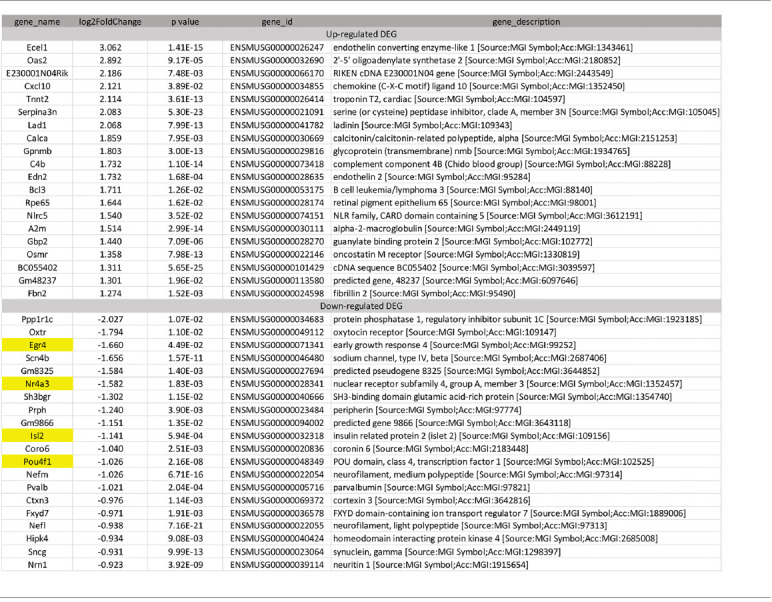

*Genes were selected by adjusted *p*-value (*p*adj) < 0.05, |log_2_FoldChange|>0 and FPKM > 0, genes highlighted in yellow are transcription factors*.

### Enrichment Analysis of DEGs Induced by Optic Neuritis

**Figure 3:**
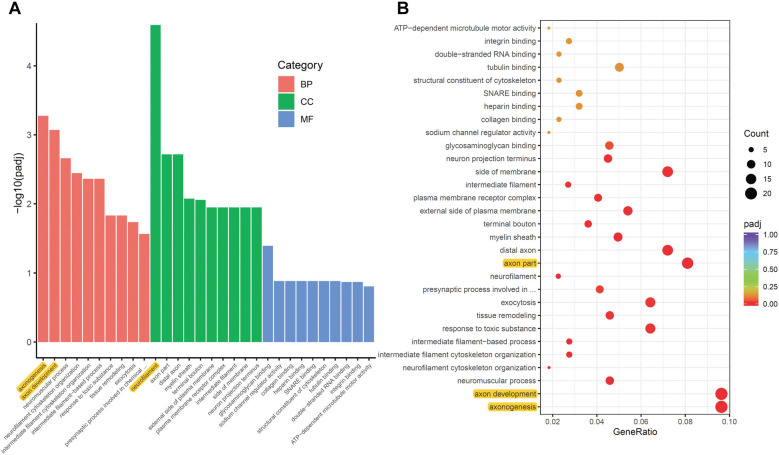
Gene ontology (GO) enrichment analysis of DEGs induced by ON. **(A)** Bar plot lists the top 10 enrichments of each category based on *p*adj value; yellow color highlights the top 3 of the most significantly enriched gene categories based on *p*adj value. **(B)** Dot plot shows the top enriched gene categories based on DEG numbers; yellow color highlights the top 3 of the enriched gene categories with most DEG numbers. BP, Biological Process; CC, Cellular Component; MF, Molecular Function. *p*adj: adjusted *p*-value.

To analyze the overall regulation effects of these DEGs induced by ON, we performed enriched gene ontology (GO) analysis. Results showed that among the three main categories of Biological Process (BP), Cellular Component (CC), and Molecular Function (MF), the top three most significantly regulated gene categories were: neurofilament, axonogenesis and axon development (Figure 3A). Axonogenesis and axon development were belonged to the main category of BP, while neurofilament was belonged to the main category of CC. As to the top 3 GeneRatio categories which contained the most count of DEGs, they were axonogenesis, axon development and axon part (Figure 3B). Axon parts also belonged to the main category of CC. However, enriched KEGG (Kyoto Encyclopedia of Genes and Genomes) pathway analysis showed no significant enriched gene categories (Supplementary Figure S1).

### Reversal of Optic Neuritis Induced DEGs by Needling Treatment

**Figure 4:**
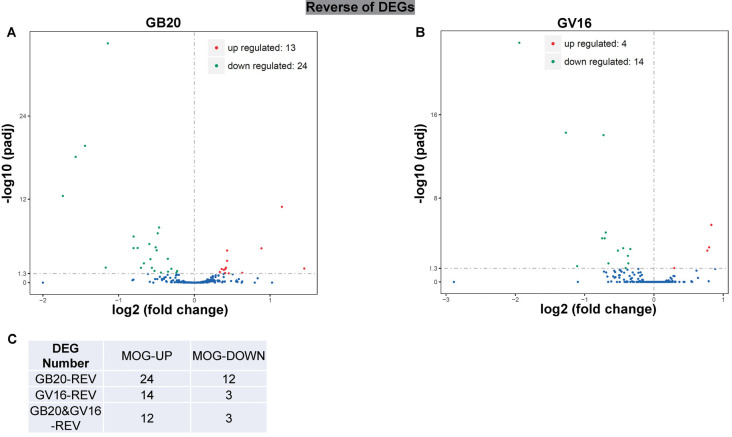
Reverse of ON-induced DEGs by needling treatments. **(A,B)** Volcano map shows the ON-induced DEGs regulated by needling treatments at acupoint GB20 and GV16, respectively. **(C)** Table shows the reversal numbers of ON-induced upregulated and down-regulated DEGs by needling treatments at acupoint GB20 and GV16, respectively. Adjusted *p*-value (*p*adj) <0.05.

To study the effect of needling treatment in mice with ON, we performed needling treatments every 3 days at acupoint GB20 or control acupoint GV16 3 weeks after MOG immunization. RNA-seq analysis showed that among the ON induced 234 DEGs, GB20 treatment significantly upregulated 13 and down-regulated 24 of total DEGs (Figure 4A), while GV16 control treatment significantly up-regulated 4 and down-regulated 14 of total DEGs (Figure 4B). Further analysis revealed that, of the ON induced 100 up-regulated DEGs (MOG vs. SHAM), 24 DEGs were reversed by GB20 treatment, while 14 were reversed by control GV16 treatment. However, 12 of the 14 reversed DEGs of GV16 treatment were already included in the 24 DEGs of GB20 (Figure 4C). The left 2 DEGs specific to GV16 control were Gfap (glial fibrillary acidic protein) and Fgf2 (fibroblast growth factor 2). Therefore, after deducting the same DEGs shared by GV16 control, there were 12 DEGs specifically down-regulated by GB20. Among the ON induced 134 down-regulated DEGs (MOG vs. SHAM), 12 DEGs were reversed by GB20 treatment, while only three DEGs were reversed by control GV16 treatment, and all the three reversed DEGs of GV16 were also shown in that of GB20, not specific to GV16 treatment (Figure 4C). After deducting the same DEGs shared by GV16 control, there were nine DEGs specifically upregulated by GB20. Together, there were totally 21 DEGs specifically reversed by GB20 treatment compared with control treatment. The reversed DEGs were listed in Table 2. DEGs specifically reversed by GB20 treatment were highlighted in green and DEGs both reversed by GB20 or GV16 treatment were highlighted in yellow (Table 2). Finally, there was one gene whose expression was further enhanced by either GB20 or GV16 treatment, which was Folh1 (folate hydrolase 1). The average FPKM values of the 234 DEGs of all groups were listed in the supplemental data (Supplementary Table S1). Mechanical stimulation at GB20 acupoint of EAE mice for 2 weeks, was resulted in reverse of total 36 ON induced DEGs (Figure 4C). Specifically, among those reversed DEGs (Table 2), Nr4a3, Sncg and Uchl1 were axonogenesis and axon development related (Soto et al., 2008; Stevanato and Sinden, 2014; Bishop et al., 2016) and down-regulated in EAE model. Tppp3 was involved in tubulin polymerization and promotion of axon regeneration (Huang et al., 2017); and Slc5a8 was involved in sodium ion transmembrane transport (Miyauchi et al., 2004). Furthermore, Nr4a3 and Sncg were among the top 20 down-regulated DEGs induced by MOG immunization (Table 1).

**Table 2:** Differentially expressed genes (DEGs) reversed by needling treatments.

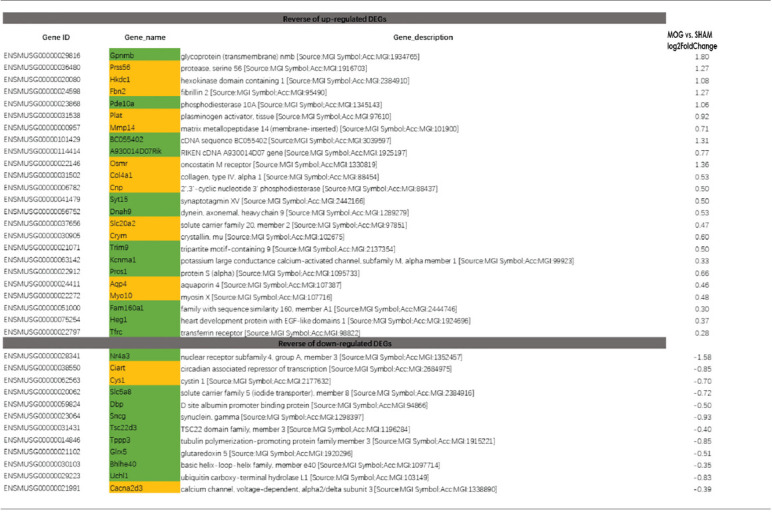

*Reversed DEGs were selected by adjusted *p*-value (*p*adj) < 0.05, |log_2_FoldChange| > 0 and FPKM > 0. The green color highlights the DEGs specifically reversed by needling treatment at GB20, while yellow highlights the DEGs reversed both by needling treatment at GB20 and GV16*.

### Enrichment Analysis of Needling Treatment

**Figure 5:**
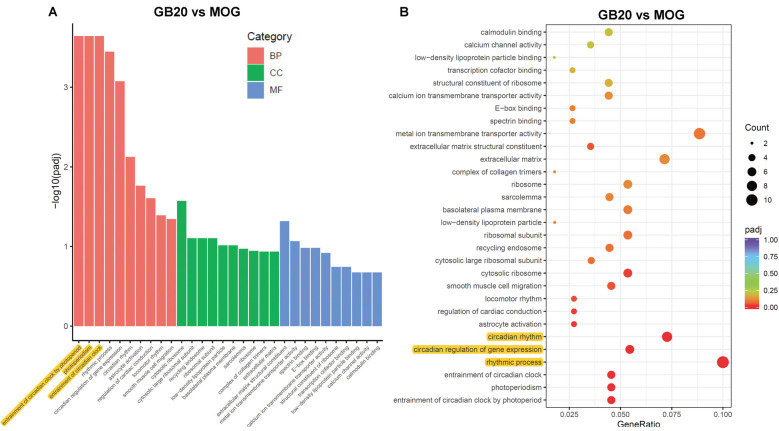
GO enrichment analysis of needling treatments at GB20. **(A)** Bar plot lists the top 10 enrichments of each category based on *p*adj value; yellow color highlights the top 3 of the most significantly enriched gene categories based on *p*adj value. **(B)** Dot plot shows the top enriched gene categories based on DEG numbers; yellow color highlights the top 3 of the enriched gene categories with most DEG numbers. BP, Biological Process; CC, Cellular Component; MF, Molecular Function. *p*adj: adjusted *p*-value.

**Figure 6:**
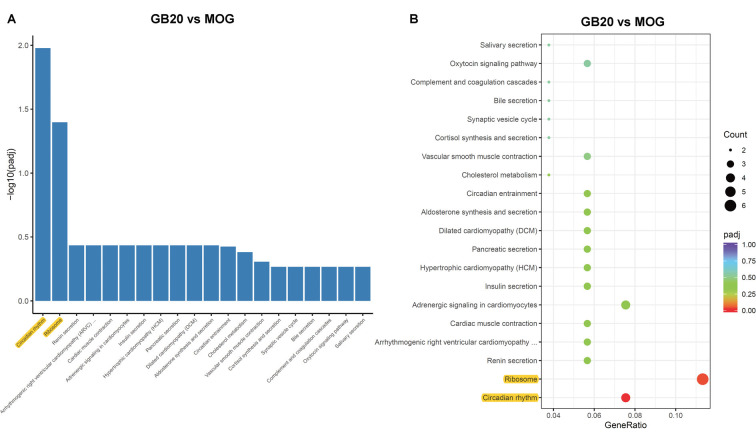
KEGG pathways analysis of needling treatments at GB20. **(A)** Bar plot lists the top enriched pathways based on *p*adj value; yellow color highlights the significant enriched pathways. **(B)** Dot plot shows the top enriched pathways based on DEG numbers; yellow color highlights the significant enriched pathways. *p*adj: adjusted *p*-value.

To analyze the overall effects of needling treatments on mice with ON, we performed enriched GO analysis comparing GB20 vs. MOG and GV16 vs. MOG. Results showed that the top 3 most significantly regulated gene categories after needling treatment at GB20 were: entrainment of circadian clock by photoperiod, Photoperiodism and Entrainment of circadian clock (Figure 5A). As to the top 3 GeneRatio categories which contained the most count of DEGs, they were Rhythmic process, Circadian rhythm, and Circadian regulation of gene expression (Figure 5B). They were all belonged to main category of BP. However, needling treatment at control acupoint GV16 showed no significant gene category enrichment (Supplementary Figure S2). Finally, KEGG pathway enrichment analysis showed that only gene categories of Circadian rhythm and Ribosome were significantly regulated (Figure 6). There were a total of 119 DEGs after needling treatment at acupoint GB20 compared with MOG group, while there were only 57 DEGs after needling treatment at control acupoint GV16 compared with MOG group (Supplementary Figure S3). The average FPKM values of the 119 DEGs induced by GB20 treatment and 57 DEGs induced by GV16 control treatment were listed in the supplemental tables (Supplementary Tables S2, S3).

## Discussion

ON is the second most frequent symptom of MS, while there is no satisfactory treatment that could prevent visual disability. In Traditional Chinese Medicine, needling is widely used in clinical trials of ocular disease treatment. However, the underlying mechanism remains unclear. In the present study, by using an EAE mouse model and retinal RNA sequencing, we demonstrate that GB20 needling can regulate the retinal transcriptome of EAE mice and reverse the expression of genes induced by ON. Many studies revealed that endoplasmic reticulum (ER) stress ER stress is closely linked to neuroinflammation. Many key molecules of unfolded protein response (UPR) pathways, such as CHOP, ATF4, BiP, and XBP1, are reported to be induced in autopsied brain specimens of MS patients and spinal cord of EAE mice (Deslauriers et al., 2011). These ER stress molecules are also induced in retina of EAE mouse (Stone and Lin, 2015; Huang et al., 2017). However, the induction of those key UPR pathway molecules is not found in the RNA sequencing analysis of EAE retina samples. This may be due to the low induction and expression level of ER stress molecules in retina with ON. In our previous study, CHOP, PERK and ATF4 were all induced in optic nerve crush model and identified by RNA sequencing analysis (Chen et al., 2019). Optic neuropathies caused by either traumatic injury such as optic nerve crush and ischemia or chronic injury such as ON all results in retinal degeneration and RGC death (Fernandes et al., 2013; Nashine et al., 2014; Huang et al., 2017; Kumar et al., 2019). Although inhibition of ER stress molecules provides neuroprotection, the manipulation of ER stress molecules does not affect the disease development of EAE mouse (Deslauriers et al., 2011; Huang et al., 2017; Yue et al., 2019). In this study, needling at GB20 specifically reversed the expression of 21 genes induced by ON, while as the same, needing treatment did not affect the clinical score of EAE development.

Among the DEGs reversed by GB20 needling treatment, we found that Nr4a3 is a transcription factor and significantly down-regulated by ON. Nr4a3 plays a critical role in pyramidal cell survival and axonal guidance (Pönniö and Conneely, 2004). Nr4a3 functions as a negative regulator of apoptotic process, and plays a neuroprotection role in oxidative stress-induced neuron death (Volakakis et al., 2010). Needling treatment at GB20 significantly increased and recovered the expression level of Nr4a3 (Table 2), indicating that mechanical stimulation at GB20 did have beneficial effects in treatment of ON.

In the previous study, we used GV16 acupoint for the first time as a control needling site for GB20 which was physically very close to the GB20 acupoint (Chen et al., 2019). In the present study, we have again proved that GV16 is the best control site for the needling treatment at GB20. Almost all the DEGs reversed by control GV16 treatment was also shown in the reversed DEG list of GB20 treatment. There are 21 DEGs that are specifically reversed by GB20 treatment (Figure 4C).

The GO enrichment analysis revealed that the most affected gene category of retina was circadian rhythm after treatment at GB20 (Figure 5B). So far, there is no report about the link between the stimulation at GB20 and the regulation of retinal circadian rhythm. Therefore, we are the first to report the connection of GB20 needling to retinal circadian rhythm regulation. There are some studies reported that acupuncture treatment with acupoints combination can upregulate the expression of circadian rhythm genes of Clock and Bmal1 in the hypothalamus (Wei et al., 2017) and the circadian rhythm genes of Per1 and Per2 in the suprachiasmatic nucleus (SCN) in insomnia rats (Guo et al., 2017), while some other research group reported that electroacupuncture treatment down-regulated the expression of Per1 and Per2 in the SCN (Hou et al., 2018). It is also reported that acupuncture treatments affect the circadian rhythm of blood pressure (Kim et al., 2012; Yang et al., 2016; Lei et al., 2017). Among the above reports, the acupoint GB20 is not included in their acupoint combinations. In this study, single needing treatment at GB20 also upregulated the gene expression of Per1 and Per2 in mouse retina, in accordance with other research groups’ reports that acupuncture treatments can affect expression of circadian rhythm genes.

The mechanism of needling at GB20 affects the regulation of retinal gene expression, is still unclear and may be extraordinarily complex, since the site of GB20 is at the back of the neck, far from the eyes (Figure 1A). A previous functional magnetic resonance imaging study revealed that needling at GB20 was able to regulate neural activity within the visual region of the brain (Li et al., 2018). It is reported that needling treatment also regulated the expression of nerve growth factor and brain-derived neurotrophic factor in retina (Pagani et al., 2006), which may regulate retinal gene expression. During neural development, neural activities also regulate neuron gene expression and formation and mature of many synapses (Hensch, 2005; Hooks and Chen, 2006; Shah and Crair, 2008; Hong and Chen, 2011; Furman and Crair, 2012; Ackman and Crair, 2014). Therefore, mechanical stimulation at GB20 may activate and transfer neural signals into the brain and then control the retinal gene express by the feedback neural signal from the brain.

## Data Availability Statement

The datasets presented in this study can be found in online repositories. The names of the repository/repositories and accession number(s) can be found below: https://www.ncbi.nlm.nih.gov/geo/, GSE148759/b.

## Ethics Statement

The animal study was reviewed and approved by the Animal Care and Use Committee of Beijing Institute of Technology and Peking University.

## Author Contributions

LM designed the experiments. JC, LZ, XG, RZ, YH, and QL performed the experiments and collected the data. LM and XL analyzed the data. HF drew the schematic image. LM prepared the figures and manuscript. All authors contributed to the article and approved the submitted version.

## Conflict of Interest

The authors declare that the research was conducted in the absence of any commercial or financial relationships that could be construed as a potential conflict of interest.

## Supplementary Material

FIGURE S1 KEGG pathways analysis of DEGs induced by ON. **(A)** Bar plot lists the top enriched pathways based on *p*adj value. **(B)** Dot plot shows the top enriched pathways based on DEG numbers. *p*adj: adjusted *p*-value.

FIGURE S2 Gene ontology enrichment analysis of needling treatments at GV16. **(A)** Bar plot lists the top 10 enrichments of each category based on *p*adj value; yellow color highlights the top 3 of the most significantly enriched gene categories based on *p*adj value. **(B)** Dot plot shows the top enriched gene categories based on DEG numbers; yellow highlights the top 3 of the enriched gene categories with most DEG numbers. BP, Biological Process; CC, Cellular Component; MF, Molecular Function. *p*adj: adjusted *p*-value.

FIGURE S3 Differentially expressed retinal genes after needling treatments at GB20 and GV16 in ON mice. **(A,B)** The volcano map shows the numbers of DEGs of retina after needling treatments at GB20 and GV16, respectively, compared with MOG immunization alone. *p*adj: adjusted *p*-value.

TABLE S1 Average FPKM values of differential expressed genes of all groups.

TABLE S2 Average FPKM values of DEGs of GB20 group compared with MOG group.

TABLE S3 Average FPKM values of DEGs of GV16 group compared with MOG group.
