# Supplementary figures and images for: Effects of Retinal Transcription Regulation After GB20 Needling Treatment in Retina With Optic Neuritis
Source: Front Integr Neurosci. 2020 Sep 29;14:568449. doi: 10.3389/fnint.2020.568449 (PMC7550785; doi:10.3389/fnint.2020.568449)

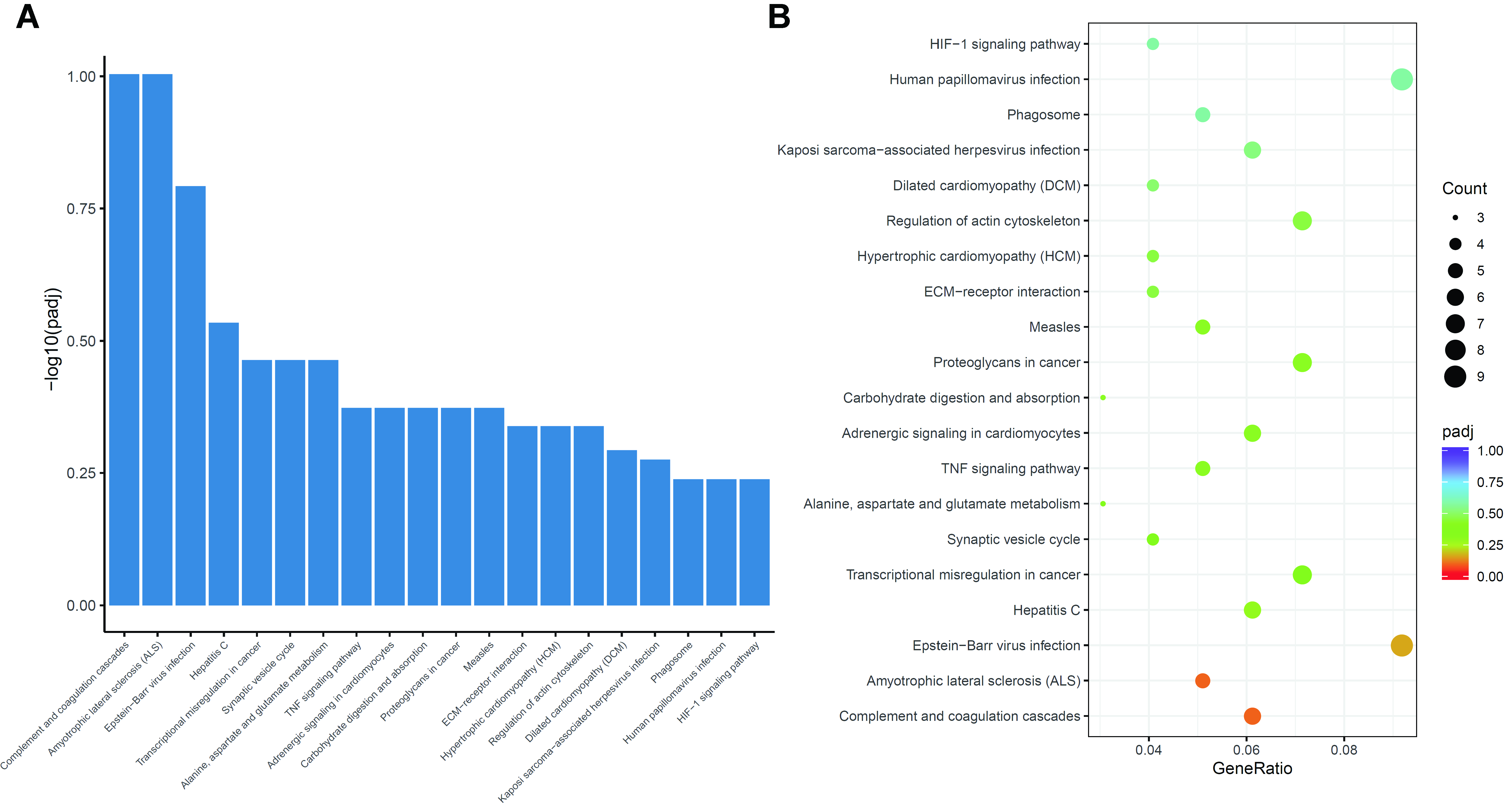

Supplement: FIGURE S1 — KEGG pathways analysis of DEGs induced by ON. (A) Bar plot lists the top enriched pathways based on padj value. (B) Dot plot shows the top enriched pathways based on DEG numbers. padj: adjusted p-value. [file Image_1.TIF]

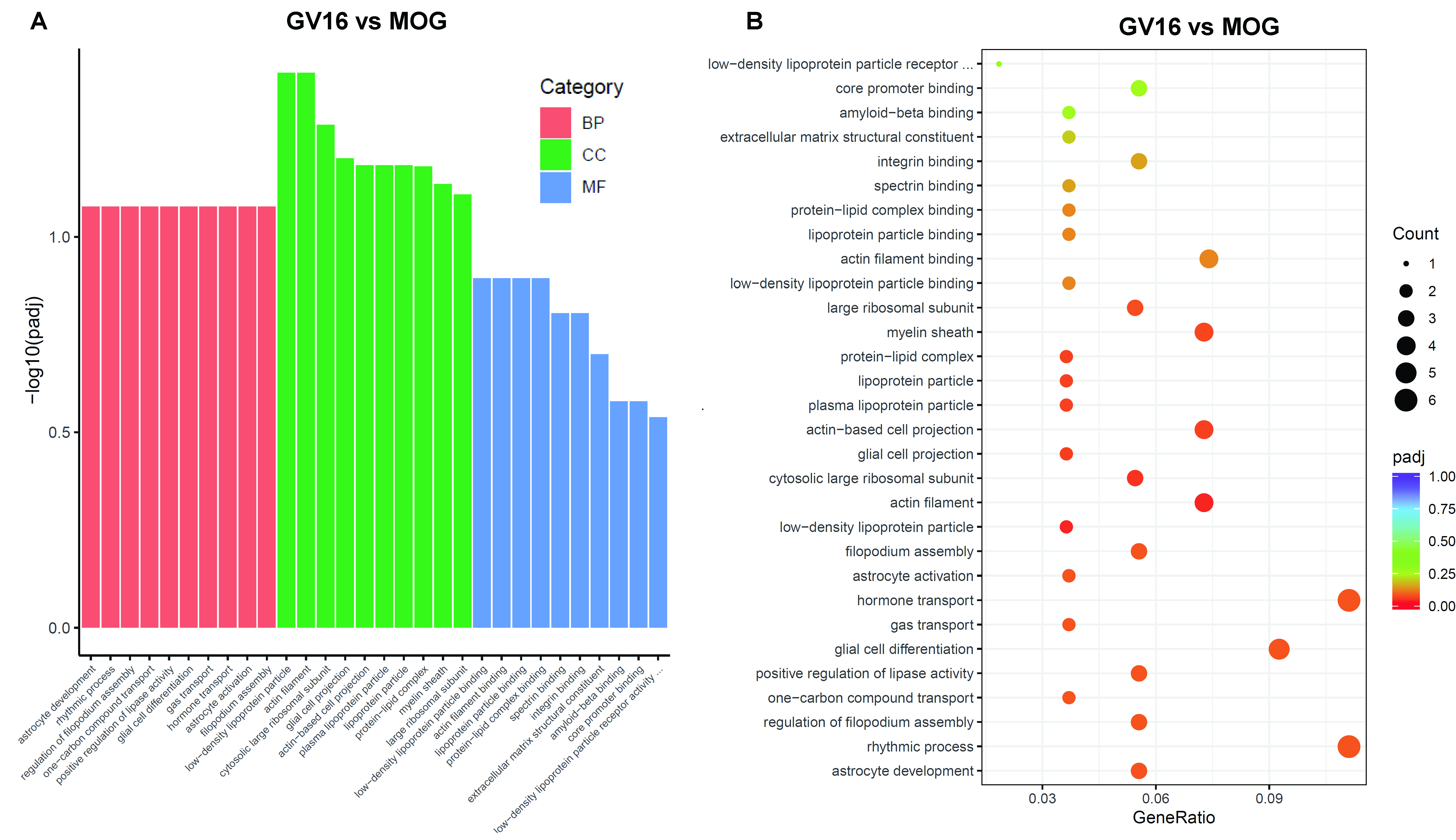

Supplement: FIGURE S2 — Gene ontology enrichment analysis of needling treatments at GV16. (A) Bar plot lists the top 10 enrichments of each category based on padj value; yellow color highlights the top 3 of the most significantly enriched gene categories based on padj value. (B) Dot plot shows the top enriched gene categories based on DEG numbers; yellow highlights the top 3 of the enriched gene categories with most DEG numbers. BP, Biological Process; CC, Cellular Component; MF, Molecular Function. padj: adjusted p-value. [file Image_2.TIF]

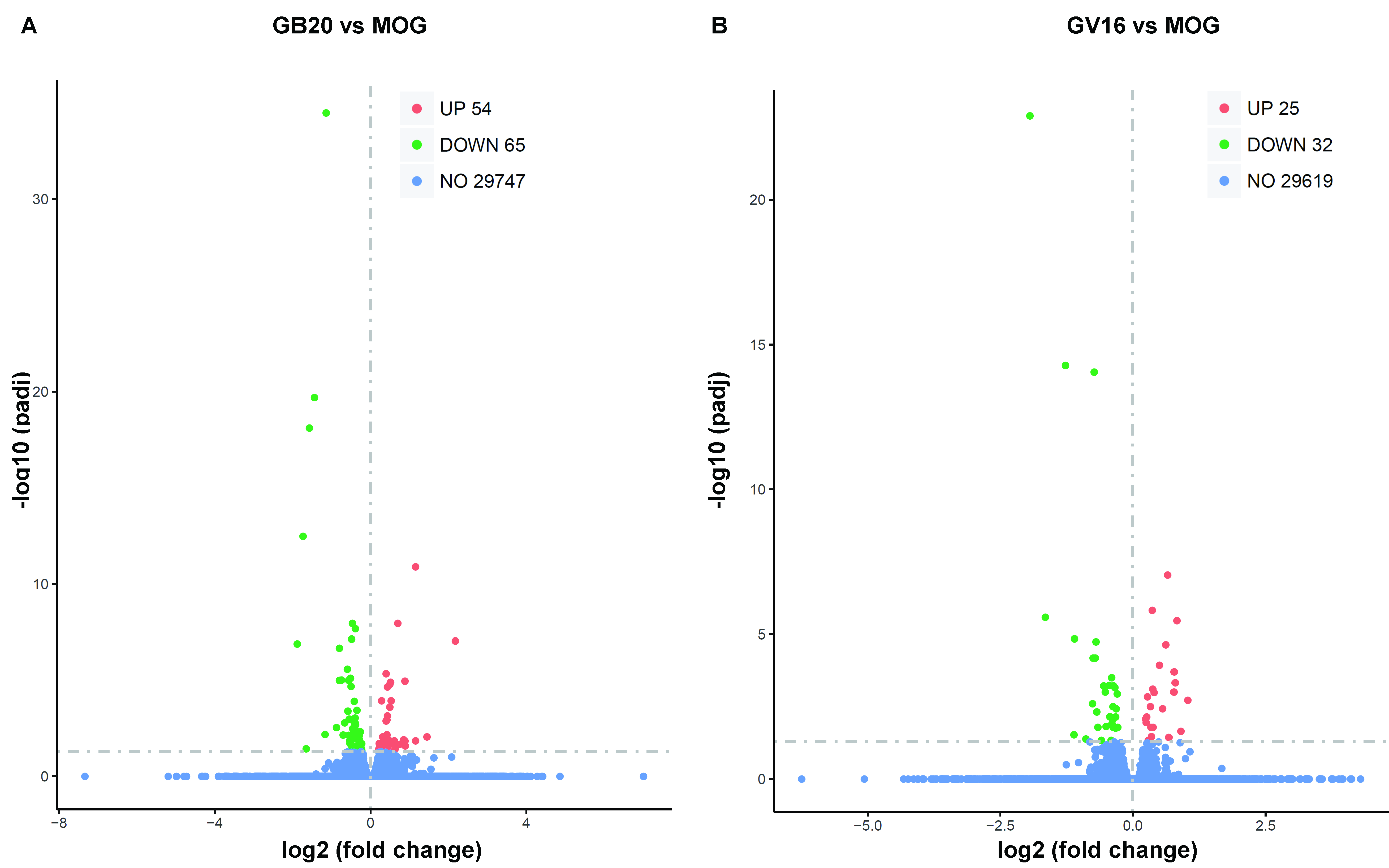

Supplement: FIGURE S3 — Differentially expressed retinal genes after needling treatments at GB20 and GV16 in ON mice. (A,B) The volcano map shows the numbers of DEGs of retina after needling treatments at GB20 and GV16, respectively, compared with MOG immunization alone. padj: adjusted p-value. [file Image_3.TIF]
